# Impact of chlorine dioxide and chlorhexidine mouthwashes on friction and surface roughness of orthodontic stainless steel wires: an in-vitro comparative study

**DOI:** 10.12688/f1000research.158974.2

**Published:** 2025-01-21

**Authors:** Shivani Apte, Divya S, Arun S Urala

**Affiliations:** 1 Department of Orthodontics and Dentofacial Orthopaedics, Manipal College of Dental Sciences, Manipal Academy of Higher Education, Manipal, Karanataka, 576104, India

**Keywords:** Mouthwash, In-Vitro study, Orthodontics, Archwires, Frictional resistance, Surface characteristics

## Abstract

**Objectives:**

Good oral hygiene measures are important for successful orthodontic treatment. They involve various types of mouthwashes which have been reported to cause alteration of mechanical properties of archwires. This study aimed to evaluate the effects of a new kind of chlorine-dioxide-containing mouthwash on the mechanical properties and surface morphology of stainless steel orthodontic archwires against the already prevalent chlorhexidine mouthwash in the market.

**Method:**

Group A – Chlorhexidine mouthwash 0.2% (study), Group B – Chlorine Dioxide mouthwash (study), and Group C – Artificial Saliva (control). 42 specimens of 5 cm long 19x25 inch SS archwires were immersed in each group equally. Post immersion, the frictional force was analyzed in the universal testing machine for each group using custom-made acrylic jigs for 10 specimens. The remaining 4 specimens from each group were sent for surface morphology evaluation using an atomic force microscope.

**Results:**

Friction resistance evaluation for the archwires revealed a mean friction of 0.011 ± 0.0056 in Group A, 0.015 ± 0.0052 in Group B, and 0.010 ± 0.0067 in Group C. Results suggested that the static friction of Group C (control group) was found to be the least when compared with the experimental groups, although not producing statistically significant values. Surface roughness of archwires compared at a 10μm range revealed a mean roughness of 19.38 ± 0.82 in Group A, 25.39 ± 7.01 in Group B, and 16.65 ± 3.07 in Group C which shows there wasn’t any statistically significant difference in the mean roughness midst the three sets.

**Conclusion:**

Chlorine dioxide and Chlorhexidine mouthwashes caused an increase in the frictional resistance of the archwires when compared to the control group. When measured at a range of 10μm the mean surface roughness did not differ across the control and the experimental groups.

## Introduction

Orthodontic treatment is the use of dental archwires and attachments to move teeth. Because the appliances can be found in the mouth and subjected to changed biological circumstances, orthodontic patients are more susceptible to gingivitis and enamel decalcification; thus, mouthwash application in addition to brushing is crucial to avoid these possible consequences.

Mouthwashes contain active agents that have been used as an adjunct to maintaining oral hygiene in patients undergoing fixed orthodontic therapy. As a result, Orthodontists should be aware of the impact of mouthwashes containing various active agents on the physical properties of various alloys to propose a suitable mouthwash to patients throughout the treatment period and use the arch wire accordingly. Researchers have struggled to develop an oral rinse that is as potent as Chlorhexidine (CHX) while having fewer side effects. Commercially accessible is a mouthwash comprising Chlorine Dioxide (ClO _2_). The active ingredient is sodium chlorite in a state of stabilized chlorine dioxide. Currently, oral rinses comprising ClO _2_ are utilized. ^1^

ClO _2_ is thought to be a good choice for usage in children since it is not oncogenic or allergic and has no influence on the taste experience. Furthermore, research has revealed that it is not as harmful to people as CHX. As a result, whereas CHX is commonly regarded as the gold standard, ClO _2_ is equally beneficial for biofilm reduction. ^2^

Previous research has shown ClO _2_ and chlorite anion to be effective bactericidal agents against majority of periodontogenic bacteria. ^3, 4^ In orthodontic patients, it has shown to be useful in reducing gingivitis and halitosis, and plaque otherwise. ^5, 6^

The biomechanical forces produced by orthodontic archwires, through brackets to cause tooth movement, are vital in clinical practice. Light, continuous forces are considered ideal for tooth movement in a regulated manner within physiologic limits, thus avoiding hyalinization, resorption, and patient discomfort. The most popular arch wire materials used today vary mainly between Stainless-steel (SS), Beta-titanium (TMA) also Nickel-Titanium (Ni-Ti) alloys. ^7^

There are constant competitive alterations in the market for newer arch wire materials and configurations to perfect the biomechanical and clinical needs necessitating the orthodontist’s need for knowledge and understanding about the same to foresee desirable treatment outcomes.

During orthodontic tooth movement, the interaction between the archwire and the bracket or ligating ligature results in static and kinetic friction, contributing to only a part of the total resistance to sliding. ^8^ The friction experienced during sliding is influenced by various factors, including the type of bracket (conventional or self-ligating), ligating force, archwire-to-bracket angulation, slot and wire dimensions and shape, repeated bracket usage, and environmental conditions such as dryness and deionization. ^9^

Additionally, rectangular wires exhibit greater frictional force than round wires, which may be due to their larger surface area. Additionally, wire size plays a role in frictional force, with smaller wires experiencing less friction than larger wires, as they have less contact with the bracket. ^10^

Stainless steel wires have remained popular since their introduction to orthodontics because of their suitable properties. It forms the stiffer arch wire that helps to bring about the key tooth movements including space closure, etc. ^11^

Despite their positive benefits, mouthwashes have the potential to alter the mechanical and surface characteristics of archwires and brackets, as well as enhance microhardness and friction. ^12^ Corrosion can significantly alter the surface properties of metals, and elevated friction during sliding within the bracket and wire can occur because of the wire’s greater surface roughness, resulting in improper distribution of load in the orthodontic appliance and, as a result, dropped efficacy of guided movement of the teeth across the archwire. ^13^

An increase in surface roughness can lead to an increase in friction, especially in dry or boundary-lubricated conditions because rougher surfaces have a higher contact area and produce more asperity (microscopic peaks and valleys) interactions, which can cause greater resistance to sliding. ^14^

According to the existing literature, studies on the effect of Chlorine Dioxide mouthwash on surface characteristics of stainless steel orthodontic archwires haven’t been conducted, therefore the purpose of this study will be towards evaluating effects of this mouthwash on the properties of stainless steel (SS) wires to formulate a mechanically useful archwire and mouthwash combination to be used during orthodontic treatment as well as to form a basis for comparison with Chlorhexidine.

## Methods

### Ethical approval

The research’s permission was acquired from the Ethics committee of Kasturba Medical College and Kasturba Hospital (IEC2: 50/2022) on 12/05/2022.

### Materials

1. Orthodontic archwires - 19 × 25 inch Stainless steel orthodontic archwires (G&H company, Libral traders)
2. Pre-adjusted Edgewise brackets - MBT prescription with a slot dimension of 0.022 inch (Forestadent)
3. Customized acrylic jig (25 cm × 7 cm)
4. Stainless steel ligature wire
5. Artificial saliva (Department of Biochemistry, Kasturba Medical College, Manipal)
6. Chlorine Dioxide Mouthwash – 500 ml
7. Chlorhexidine Mouthwash (0.2%) – 500 ml
8. Borosil ^®^ petri dishes for keeping the wires immersed
9. Universal testing machine with load weight (10 N) – Dental Materials, Manipal
10. Atomic Force Microscope – Innovation Centre, MIT

## Method

### Preparation of wire samples and immersion process in test groups

**Figure 1.**
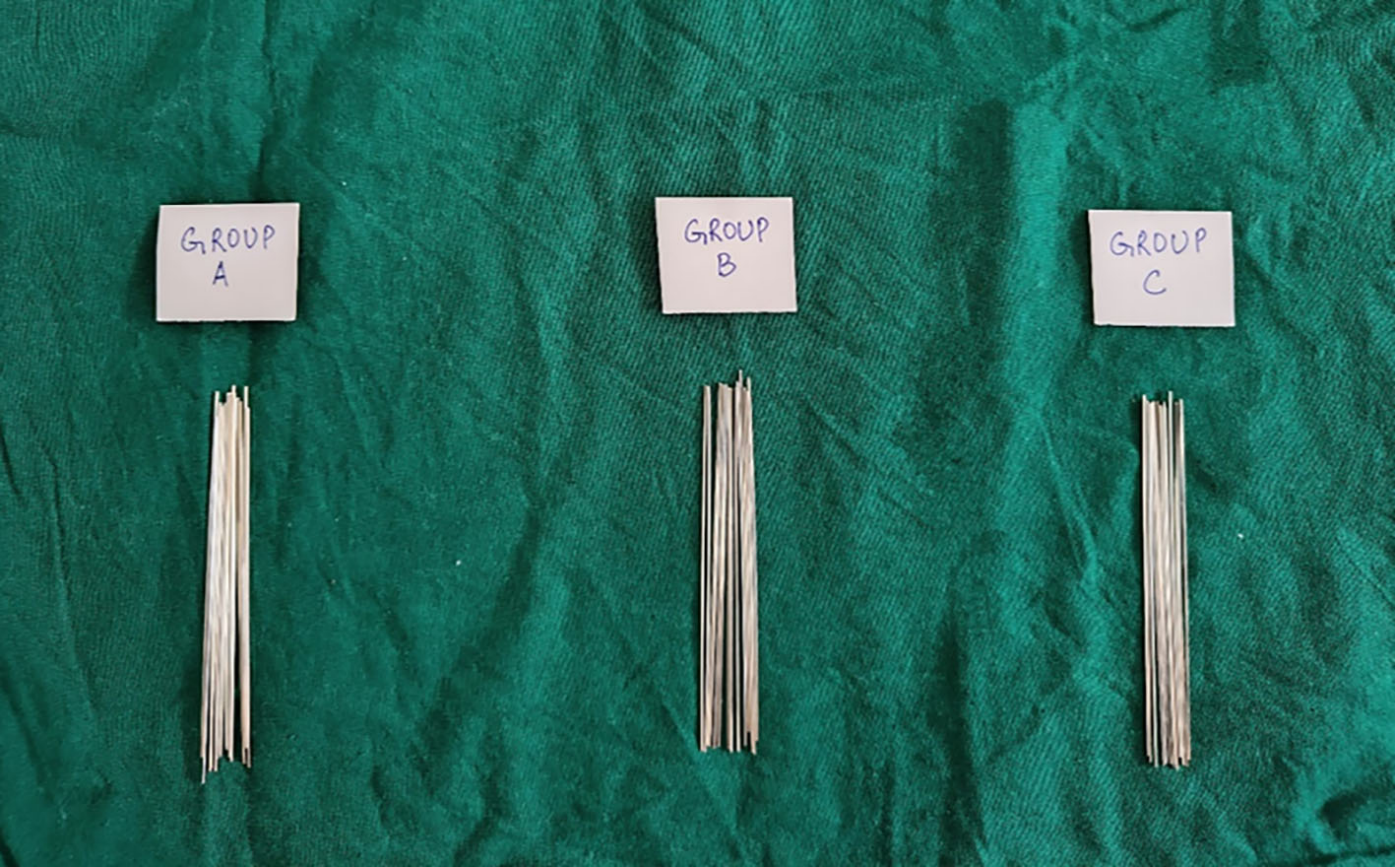
19 × 25 inch stainless steel archwires.

**Figure 2.**
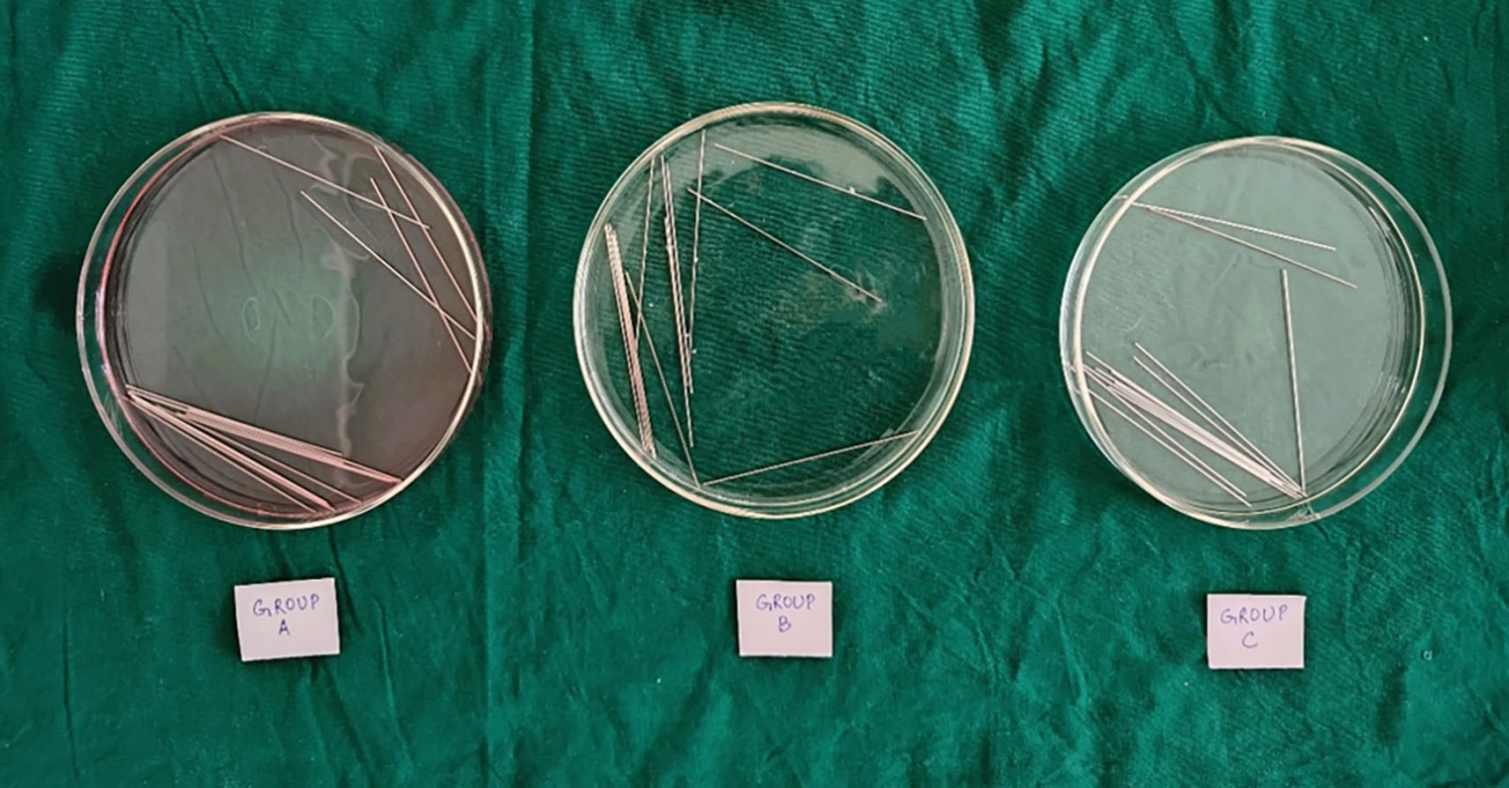
Wire samples in different immersion groups.

• Experimental mouthwashes i.e. Chlorine Dioxide mouthwash (ClO _2_) and Chlorhexidine mouthwash (CHX) were procured. 126 upper stainless-steel metal brackets with 0.022-inch slot sizes were selected. 0.019 × 0.025 inch straight rectangular stainless-steel arch-wires of a sample size of 42 were used ( Figure 1).
• Sample size calculation:
  Effect size: 0.5
  Power of the study: 0.8
  α Error: 0.05
  No. of groups: 3
  Calculated sample size: 42
  Per group: 14
• The wires were cut, and experimental units were immersed in 0.2% Chlorhexidine mouthwash (Group A) and Chlorine Dioxide mouthwash (Group B) respectively, for 30 seconds twice daily for 3 months. During the time interval of each immersion apart from the mouthwash immersion, the experimental units were immersed in artificial saliva ( Figure 2).
• The control units were immersed in artificial saliva (Group C), obtained from the Department of Biochemistry for 3 months. ^15, 16^ 14 specimens of wires were immersed in an individual 20-ml Borosil petri dish per group in 3 groups ( Figure 2). After a period of 3 months, the experimental and control specimens were removed from their respective solutions and rinsed with distilled water for evaluation.

### Frictional force evaluation

**Figure 3.**
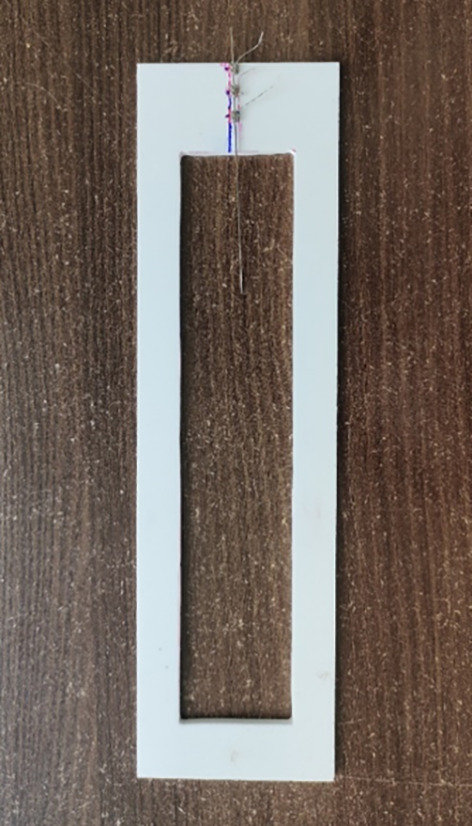
Bracket-wire assembly on custom jig.

**Figure 4.**
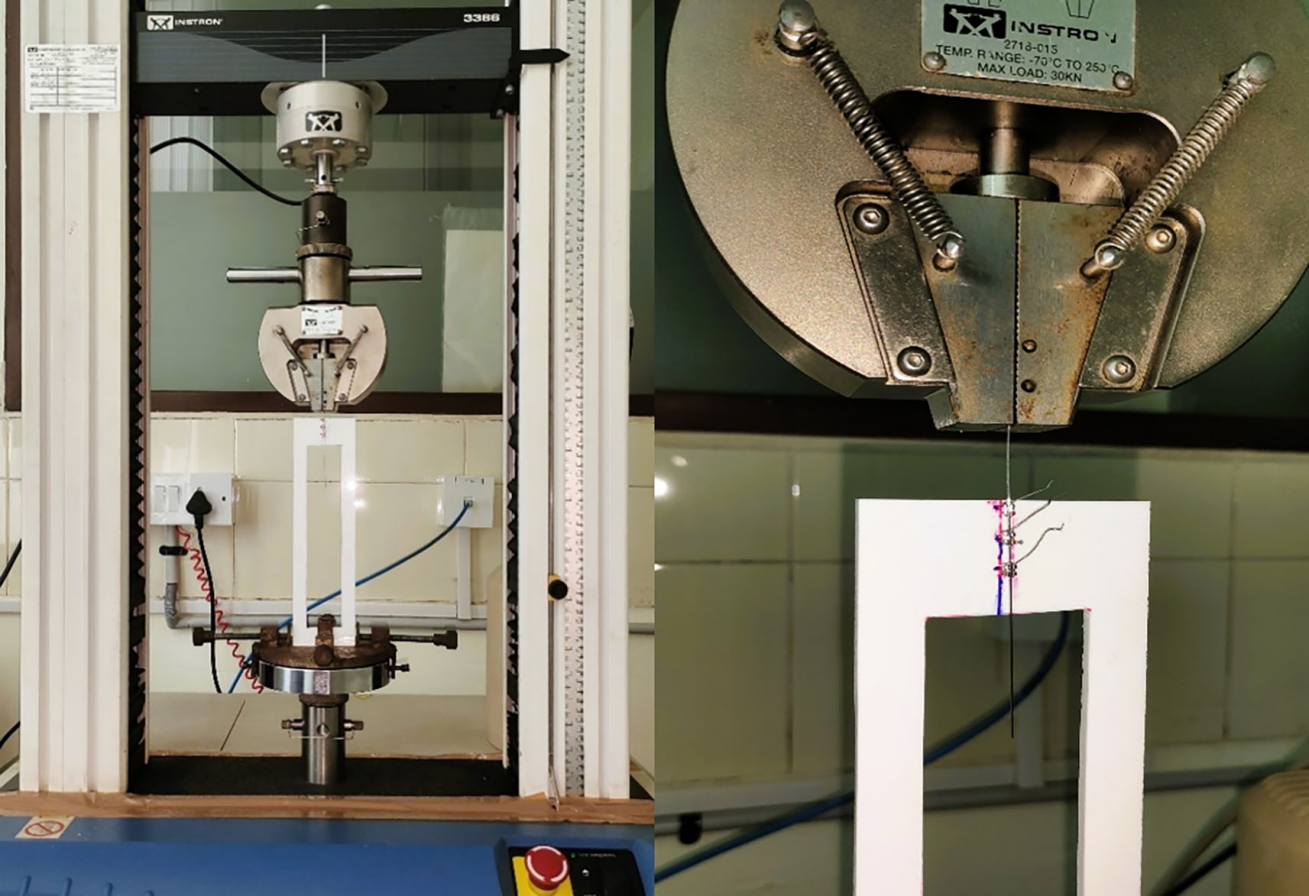
Universal testing machine with the customized jig and load setup.

• Frictional force was measured using a Universal Testing Machine in dry conditions without thermocycling of wire samples after immersion process. ^17^
• 10 Samples from each group were tested. A customized jig was designed for grasping wires during testing. The brackets were bonded on the jigs and ligation between the bracket and wire was with a stainless-steel ligature wire during frictional force evaluation which was standardized with 11 twists per bracket using a Mathew needle holder (GDC) ( Figure 3).
• A load cell was calibrated between 0 and 10 N, and the archwire was drawn through the bracket at a crosshead speed of 10 mm/min over a 5-mm section of the arch-wire ( Figure 4).
• After every test, the bracket-wire grouping was removed, and a new assembly was.placed. The recorded data was statistically analyzed to determine significant differences between the one type of archwire immersed in two test environments ( Figure 4).

### Archwire surface morphology evaluation

**Figure 5.**
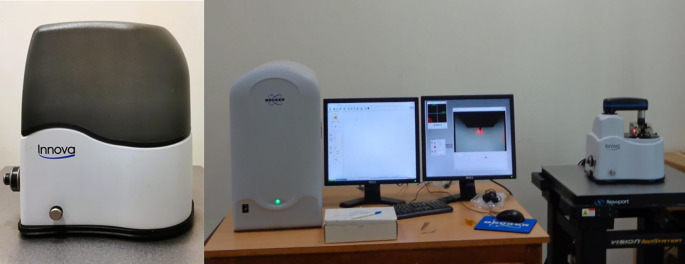
Atomic Force Microscope with the computer software.

**Figure 6.**
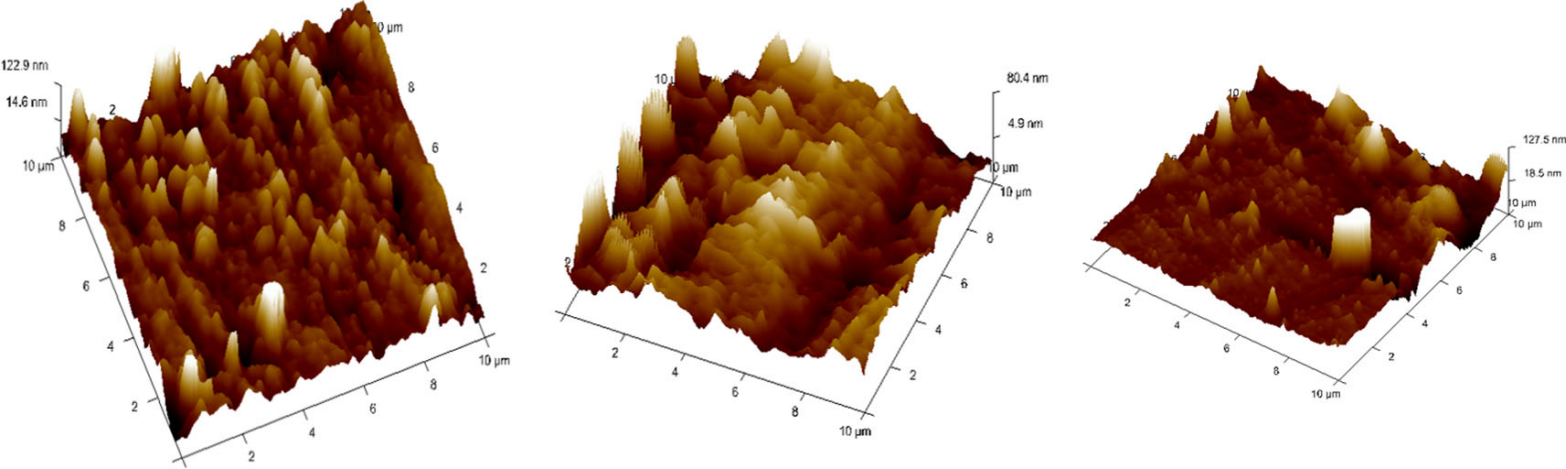
Three-dimensional images for external surfaces of stainless steel archwires obtained with Atomic Force Microscope (AFM) and Surface roughness measured at a 10μm range for Group A (CHX).

**Figure 7.**
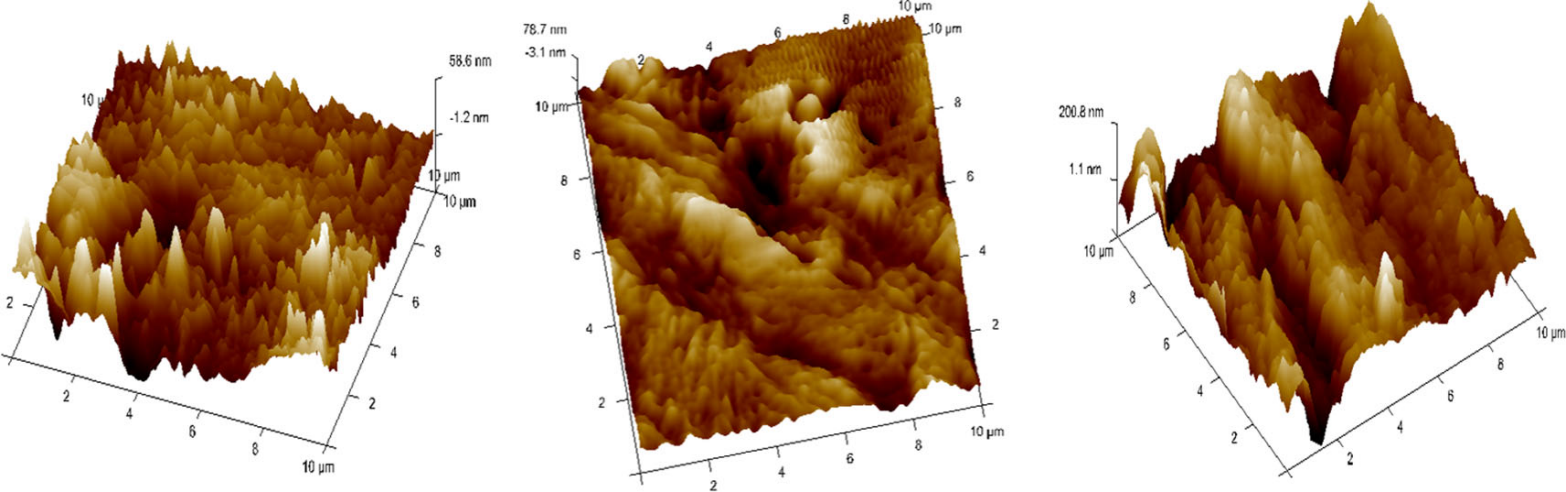
Three-dimensional images for external surfaces of stainless steel archwires obtained with Atomic Force Microscope (AFM) and Surface roughness measured at a 10μm range for Group B (ClO _2_).

**Figure 8.**
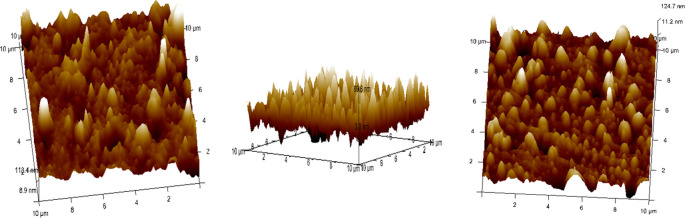
Three-dimensional images for external surfaces of stainless steel archwires obtained with Atomic Force Microscope (AFM) and Surface roughness measured at a 10μm range for Group C (Artificial Saliva).

• The remaining 4 samples from each group of specimens were cleaned with 95% ethanol before Atomic Force Microscope (AFM) observations to be done in dry condition.
• The surface topography and roughness (Ra) of the stainless steel (SS) arch-wires after immersion in the Chlorhexidine, Chlorine dioxide, and artificial saliva were evaluated using an AFM ^18^ ( Figure 5).
• Each wire sample that was selected was cut to a length of 1 cm using a distal end cutter. At a scanning rate of 10 Hz, scanning was done while in the air. Four specimens per group were used to quantify surface roughness, and each specimen produced a total of three measurements over a 10 × 10-μm region ( Figures 6, 7, 8). The software provided with the AFM was used to evaluate the wire’s external surface.
• The Ra, Rq, and Rmax values were obtained using the software.
• The recorded data of surface roughness after the immersion test were statistically measured.

### Statistical analysis

Data was analysed using the statistical.package SPSS 26.0 (SPSS Inc., Chicago, IL) and the level of significance was set at p<0.05. To evaluate the mean and standard deviation of each group, descriptive statistics were used. The normality of the data was assessed using the Shapiro-Wilkinson test. One way ANOVA was used in inferential statistics to determine the difference within the group (3 groups) followed by Bonferroni Post-hoc Test. Pearson Correlation Test was used for correlation analysis. Data was presented using tables and bar and plot graphs.

## Results

**Figure 9.**
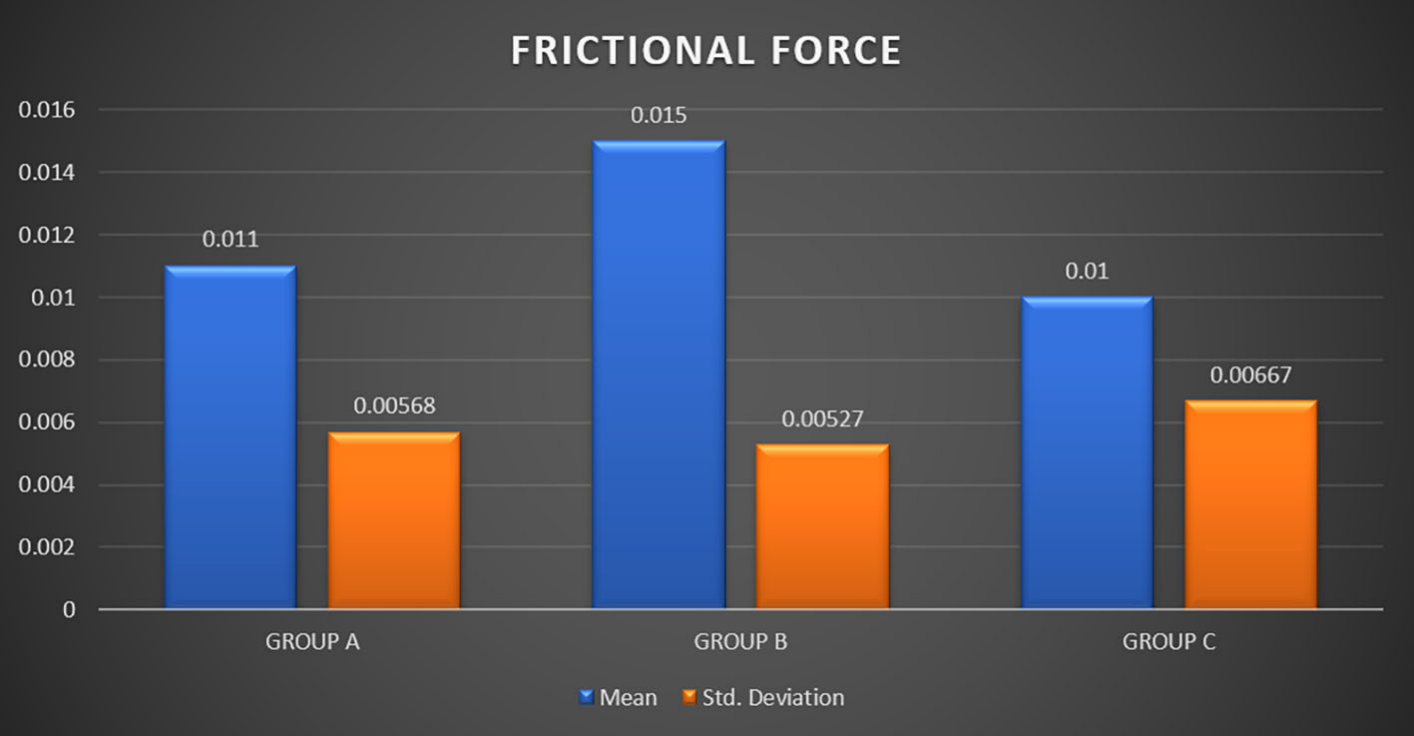
Graph 1.

Table 1 and Figure 9 provide the results of a one-way analysis of variance (ANOVA) conducted to compare the frictional force among three groups (Groups A, B, and C) corresponding to different immersion solutions i.e. Chlorhexidine, Chlorine Dioxide, and artificial saliva respectively. Regarding ‘FRICTIONAL FORCE’ Inter-group analysis by ONE WAY ANOVA did not report a significant difference (p > 0.05).

**Table 1.** Comparison of frictional force using one way ANOVA.

ANOVA					
Frictional force					
	Sum of Squares	df	Mean Square	F	Sig.
Inter-Group	.000	2	.000	2.011	.153
Intra-Group	.001	27	.000		
Total	.001	29			

* p < 0.05 is statistically significant (Shapiro Wilkinson test, p > 0.05).

Table 2 presents the results of post hoc pairwise comparisons using the Bonferroni correction method for multiple comparisons. These comparisons are conducted to determine whether there are significant differences in mean frictional force between each pair of groups (A, B, and C) after conducting a one-way ANOVA.

**Table 2.** Post hoc test for multiple comparison.

Multiple comparisons						
Dependent variable: Frictional force						
Bonferroni						
(I) V5	(J) V5	Mean Difference (I-J)	Std. Error	Sig.	95% Confidence Interval	
					Lower bound	Upper bound
GROUP A	GROUP B	-.00400	.00264	.424	-.0107	.0027
	GROUP C	.00100	.00264	1.000	-.0057	.0077
GROUP B	GROUP A	.00400	.00264	.424	-.0027	.0107
	GROUP C	.00500	.00264	.207	-.0017	.0117
GROUP C	GROUP A	-.00100	.00264	1.000	-.0077	.0057
	GROUP B	-.00500	.00264	.207	-.0117	.0017

These results suggest that there are no significant differences in mean frictional force between any pair of groups (A, B, and C) based on the Bonferroni-corrected post hoc pairwise comparisons. The p-values for all comparisons are greater than the chosen significance level (>0.05), indicating that the observed differences are not statistically significant.

**Figure 10.**
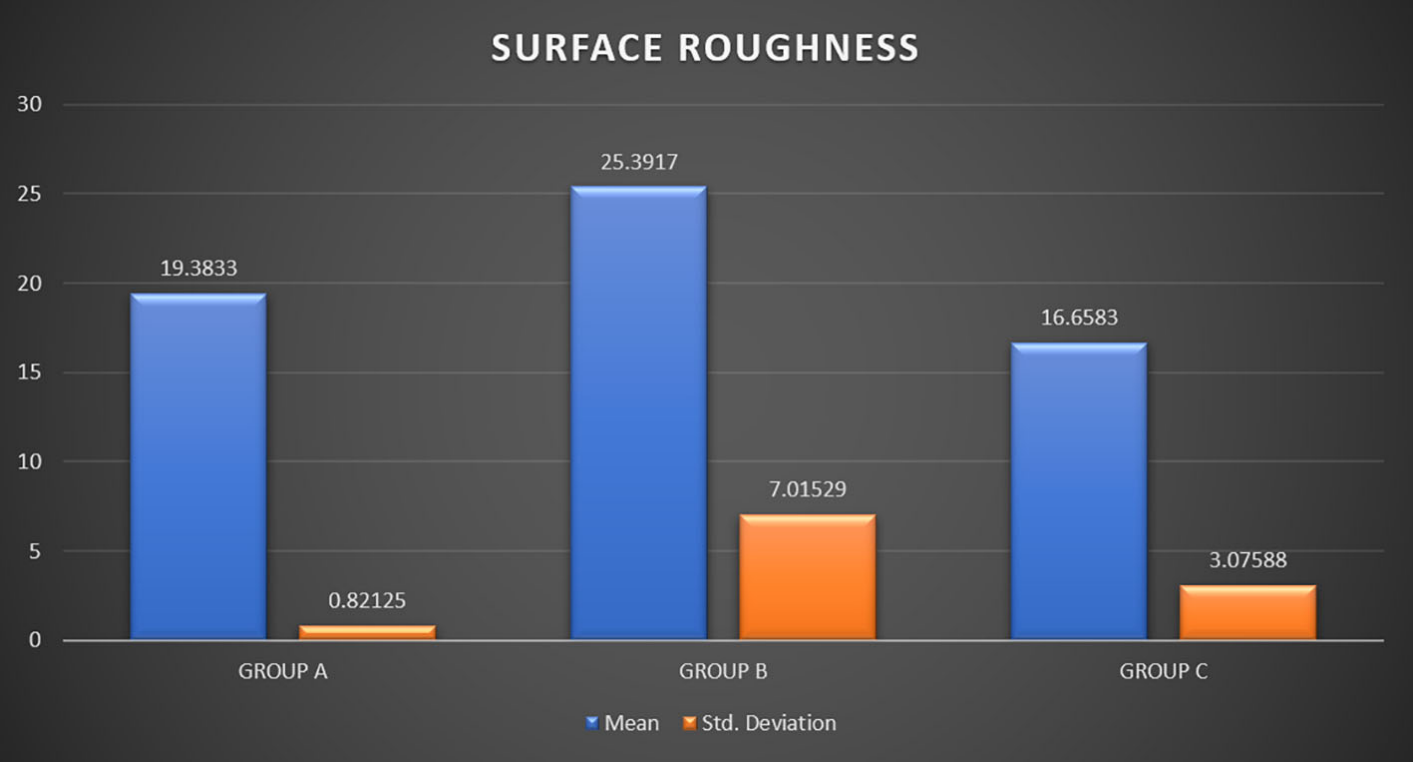
Graph 2.

Surface roughness was analyzed for the arch-wires at 3 separate regions with a range of 0-10μm and an average of three values was taken to examine the data. A one-way ANOVA was then done to analyze if the mean roughness was the same across all the groups. There was no significant difference concerning mean surface roughness among the three study groups (p = 0.056). Table 3 and Figure 10 depict the effects mouthwashes and artificial saliva had on the surface roughness of arch-wires compared at a 10 μm range.

**Table 3.** Comparison of surface roughness using one way ANOVA.

ANOVA					
Surface roughness					
	Sum of Squares	df	Mean Square	F	Sig.
Between Groups	159.729	2	79.865	4.037	.056
Within Groups	178.049	9	19.783		
Total	337.779	11			

Post hoc tests for multiple comparisons were done using the Bonferroni correction method ( Table 4). There is no statistically significant difference between any of the pair group comparisons in terms of surface roughness (p > 0.05).

**Table 4.** Post hoc test for multiple comparison.

Multiple comparisons						
Dependent variable: Surface roughness						
Bonferroni						
(I) V6	(J) V6	Mean Difference (I-J)	Std. Error	Sig.	95% Confidence Interval	
					Lower bound	Upper bound
GROUP A	GROUP B	-6.00833	3.14510	.265	-15.2339	3.2173
	GROUP C	2.72500	3.14510	1.000	-6.5006	11.9506
GROUP B	GROUP A	6.00833	3.14510	.265	-3.2173	15.2339
	GROUP C	8.73333	3.14510	.065	-.4923	17.9589
GROUP C	GROUP A	-2.72500	3.14510	1.000	-11.9506	6.5006
	GROUP B	-8.73333	3.14510	.065	-17.9589	.4923

## Discussion

In orthodontic therapy, dental archwires and attachments are used for moving teeth. Stainless steel (SS) is often used in orthodontic treatment due to its superior resistance to corrosion and protective oxide coating on its surface. Because the appliances are in the mouth and exposed to changing biological conditions, orthodontic patients are more susceptible to gingivitis and enamel decalcification. To prevent these potential dangers, mouthwash should be used in conjunction with brushing. Chromium oxide serves as the protective layer for stainless steel wires. However, this protective layer is prone to mechanical and chemical rupture as measured by frictional force and surface characteristics following immersion in various solutions. ^19^

Chlorine dioxide mouthwash is a type of oral rinse that uses chlorine dioxide as its active ingredient. It is known for its ability to help reduce bad breath, as it is effective at neutralizing volatile sulfur compounds that are often responsible for halitosis. The use of chlorine dioxide in mouthwash is seen as beneficial due to its oxidizing properties, which can disrupt the cell walls of microorganisms, leading to their destruction. When considering its effects on dental materials, like stainless steel archwires commonly used in orthodontics, it’s important to evaluate whether the oxidative nature of chlorine dioxide could cause corrosion or any other negative impact on the materials’ integrity or function. The study of these interactions is valuable for orthodontic care and can inform recommendations for oral hygiene practices in individuals undergoing orthodontic treatment.

In general, stainless steel is resistant to many types of chemical exposures, but certain substances could cause problems such as surface pitting, discoloration, or even corrosion. Given the oxidizing nature of chlorine dioxide, there is a possibility that prolonged exposure or high concentrations could have some impact on the surface of stainless-steel archwires.

Previous research has not looked at the influence of chlorine dioxide-containing protective agents on the surface.roughness of stainless-steel archwires or the frictional resistance between orthodontic metal brackets and archwires. In this research, we intended to determine how chlorine dioxide-containing mouthwash affected the frictional force and surface roughness of stainless-steel orthodontic archwires, given the assumption that these properties would not alter much. This would aid in the development of safe methods for those wearing braces who want to use chlorine dioxide mouthwash in their oral hygiene routine.

The present study assessed static friction since the sliding motion of teeth down an archwire is not uninterrupted but occurs in a sequence of brief leaps or jumps. Thus, static friction is more critical thank inetic friction since that must be overcome every time there is movement of teeth. ^3^

Concerning friction, a greater amount of it for the chlorine dioxide group in comparison with the Chlorhexidine group was seen, but not significantly (p < 0.05). In a different investigation conducted by Alwafe et al., ^20^ wires placed in a fluoride-containing mouth rinse had the greatest mean coefficient of friction and roughness of the surface, after herbal mouthwash, and the lowest value for artificial saliva. Several studies have investigated the influence of fluoridated mouth rinses on surface texture ^12, 16, 20^ and the wear of orthodontic archwires and brackets. Although, the impact of chlorine dioxide-comprising mouthwash in this area hasn’t been well studied. In a prior in-vitro investigation, ^18^ the influence of chlorhexidine on the surface characteristics as well as the friction between orthodontic wires and brackets was assessed. The findings demonstrated that surface roughness of archwires and the frictional force in between two varieties of orthodontic wires made of stainless-steel and NiTi alloy metals and stainless-steel brackets were not substantially impacted by a 1.5-hour immersion in mouthwashes containing 0.2% chlorhexidine. However, in contrast to the Persica mouthwash, another investigation over the surface.morphology of orthodontic archwires determined that mouthwashes containing 0.12% chlorhexidine and 0.12% peroxide had a greater pitted appearance on stainless-steel and NiTi wires, respectively. ^21^ Given that chlorhexidine molecules are known to form bonds with the oral mucous membrane and teeth, outcomes of any in vitro investigation may differ from those of an in vivo study. Laboratory studies that execute the sliding mechanism amid a lack of moisture may also overstate the resistance to friction as saliva acts as a lubricant.

When determining surface roughness, a recent study showed that asperities were found to be more pronounced in SS arch wires and found to be less in deionized and ionized conditions. The effective area of contact between two surfaces is determined by these asperities that bear the entire load between the surfaces. ^22^

The study’s overall findings indicate that, although not statistically important, the frictional force and roughness of stainless-steel wires immersed in chlorine dioxide were higher; additionally, SS wires immersed in chlorhexidine did not exhibit significantly different surface behavior from that of the artificial saliva category. The surface information in our results was in line with those of Bundy K ^23^ and Rondelli G, ^24^ which demonstrated the high corrosion resistance of orthodontic wires in a variety of solutions such as Ringer, artificial saliva, and NaCl had a high corrosion resistance. ^25^ From the perspective of film breakdown, titanium alloys used in these solutions have a stronger resistance to corrosion than stainless steel alloys. Although not significantly different from one another, wires submerged in ClO _2_ were found to have the most surface flaws in this investigation.

In our study, conducting post hoc tests despite a non-significant ANOVA result is justified for exploratory and descriptive purposes. These tests allow us to identify potential trends or localized differences between specific group pairs, which may not have been evident in the overall ANOVA result, helping to generate hypotheses for future research. Additionally, they provide detailed pairwise comparisons to summarize group relationships, offering a clearer picture of the data's structure. This approach also contextualizes the non-significant ANOVA result, revealing whether observed differences are uniformly small or vary among groups. While these findings are exploratory and not confirmatory, they provide valuable insights into our dataset and inform potential directions for further investigation.

Dartar Oztan et al. evaluated the level of stainless-steel deterioration in mouthwashes containing EDTA, chlorhexidine (0.2%), and NaCl (5.25%). The findings indicated that there was a degree of corrosion in the stainless steel files, particularly pitting corrosion with the chlorhexidine solution. ^26^ Dental rinses with chlorhexidine as an ingredient may be recommended as harmless preventive measures, according to research by Hosseinzadeh Nik et al. ^18^

According to the present investigation, it was determined that the stainless-steel wires immersed in mouthwash containing chlorine dioxide had the highest levels of friction and surface roughness, albeit not significantly. It also found that stainless steel wires are detrimentally affected by chlorhexidine.

The methodology cannot perfectly replicate the actual clinical setting, as with any in vitro study. It’s also important to remember that the resistive frictional forces found in our study differed significantly from those that were exerted during orthodontic movement. The duration of exposure in our investigation was rather short, and the experimental setting might not accurately represent the complex oral environment. A further constraint of this research is that it has not examined other varieties of orthodontic archwires, like Ni-Ti and beta-titanium wires, so it isn’t possible to generalize its findings to them. Future research should aim to expand upon these findings by conducting in vivo studies to evaluate the effects of Chlorine dioxide-containing mouthwash on orthodontic materials in a more clinically relevant setting. Additionally, investigations into the effects of other mouthwash formulations and their potential interactions with orthodontic materials would provide valuable insights into optimizing patient care.

## Conclusion

In conclusion, our study suggests the following:

1. Chlorine dioxide-containing mouthwash does not significantly affect the frictional force and surface roughness of stainless steel orthodontic archwires under the conditions tested.
2. Chlorine dioxide may be a viable adjunct to orthodontic treatment. However, further research, particularly in vivo studies, is necessary to validate these findings and ascertain the long-term effects of Chlorine dioxide on orthodontic materials.

## Clinical significance

Evaluating the effects of this mouthwash on the properties of stainless steel (SS) wires will help researchers formulate a mechanically useful archwire and mouthwash combination to be used during orthodontic treatment as well as to establish a future base for comparison of Chlorine Dioxide mouthwash with gold standard Chlorhexidine mouthwash.

## Ethical approval

Before commencing the study, clearance was sought from the Kasturba Medical College and Kasturba Hospital Institutional Ethics Committee, Manipal, Karnataka – IEC2: 50/2022 on 12/05/2022.

## Informed consent

Not applicable as it is an in vitro study.

## Data Availability

Figshare: Impact of chlorine dioxide and chlorhexidine mouthwashes on friction and surface roughness of orthodontic stainless steel wires: an in vitro comparative study. Doi: https://doi.org/10.6084/m9.figshare.27646026.v1 ^27^ This project contains following underlying data:
1. Excel.xlsx 2. Word document.docx Data are available under the terms of the Creative Commons Attribution 4.0 International license (CC-BY 4.0).
