# Supplementary material for: Impact of chlorine dioxide and chlorhexidine mouthwashes on friction and surface roughness of orthodontic stainless steel wires: an in-vitro comparative study
Source: F1000Res. 2025 Jan 21;13:1442. Originally published 2024 Nov 28. [Version 2] doi: 10.12688/f1000research.158974.2 (PMC11754953; doi:10.12688/f1000research.158974.2)
Supplement: Peer review history

# Reviewer report: Reviewer response for version 2

**DOI:** 10.5256/f1000research.176592.r361339

**Published:** 2025-01-22

**Authors:** Sarmad S. Salih Al Qassar

**Affiliations:** 1 University of Mosul, Mosul, Iraq

The Authors addressed my concerned successfully,  

 The manuscript now is ready for indexing.

 I suggest accepting in this version.

Is the work clearly and accurately presented and does it cite the current literature?

Partly

If applicable, is the statistical analysis and its interpretation appropriate?

Partly

Are all the source data underlying the results available to ensure full reproducibility?

Partly

Is the study design appropriate and is the work technically sound?

Yes

Are the conclusions drawn adequately supported by the results?

Yes

Are sufficient details of methods and analysis provided to allow replication by others?

Yes

Reviewer Expertise:

I am working on the dental material research, polymers and 3D analysis beside I have some research regarding friction.

I confirm that I have read this submission and believe that I have an appropriate level of expertise to confirm that it is of an acceptable scientific standard.

# Reviewer report: Reviewer response for version 1

**DOI:** 10.5256/f1000research.174636.r344504

**Published:** 2024-12-23

**Authors:** Sarmad S. Salih Al Qassar

**Affiliations:** 1 University of Mosul, Mosul, Iraq

I would like to express my sincere gratitude for considering me as a reviewer for the manuscript titled *Impact of Chlorine Dioxide and Chlorhexidine Mouthwashes on Friction and Surface Roughness of Orthodontic Stainless Steel Wires: An In-Vitro Comparative Study*. The topic is indeed quite fascinating, particularly the exploration of chlorine dioxide as a mouthwash and its effect on the friction of stainless steel wires. The manuscript thoroughly discusses the role of these mouthwashes in influencing both the surface roughness of the wire and the friction at the bracket-wire interface. While the manuscript is generally well-designed and well-written, I have identified a few areas that could be addressed to enhance its quality.

 In the Abstract section, I suggest that statistical terms be omitted from the conclusion, as they are more appropriate for the results section.

 In the Introduction, there are several lengthy sentences, and the number of references cited appears to be somewhat limited. I recommend revising these sentences for clarity and strengthening the reference list to provide more comprehensive context.

 The Methodology section would benefit from further details regarding the effects of thermocycling on friction. Additionally, it would be helpful for the authors to clarify how the sample size was calculated.

 I also suggest that the authors specify the testing conditions more clearly, particularly in regard to whether the results were obtained in dry or wet conditions. Furthermore, it would be beneficial to consider the ligation adjustment for all samples.

 Regarding statistical analysis, I would like to inquire about the utility of conducting a post hoc statistical test for the non-significant results from the ANOVA test.

 Moreover, the Results section seems to be missing the correlation results that were referenced in the Methodology section, and I believe these should be included for completeness.

 Finally, I recommend that the authors include more recent references to update the manuscript with the latest information on friction and surface roughness. For instance, the following studies might be valuable additions: 

 [Ref 1] and [Ref 2]

 Thank you once again for the opportunity to review this manuscript. I believe that by addressing these points, the authors will be able to further strengthen their work.

 Sincerely,

Is the work clearly and accurately presented and does it cite the current literature?

Partly

If applicable, is the statistical analysis and its interpretation appropriate?

Partly

Are all the source data underlying the results available to ensure full reproducibility?

Partly

Is the study design appropriate and is the work technically sound?

Yes

Are the conclusions drawn adequately supported by the results?

Yes

Are sufficient details of methods and analysis provided to allow replication by others?

Yes

Reviewer Expertise:

I am working on the dental material research, polymers and 3D analysis beside I have some research regarding friction.

I confirm that I have read this submission and believe that I have an appropriate level of expertise to confirm that it is of an acceptable scientific standard, however I have significant reservations, as outlined above.

## References

[1] : Assessment of the influence of metal ions released from the fixed orthodontic appliances on the static friction and surface topography of stainless steel and I archwires: An in-vitro study. *J Orthod Sci* .2023;12: 10.4103/jos.jos_58_23 82 10.4103/jos.jos_58_23 38234657 PMC10793865

[2] : Comparative Evaluation of Frictional Resistance Between Different Types of Ceramic Brackets and Stainless Steel Brackets With Teflon-Coated Stainless Steel and Stainless Steel Archwires: An In-Vitro Study. *Cureus* .2022;14(4) : 10.7759/cureus.24161 e24161 10.7759/cureus.24161 35586355 PMC9107794

# Response

**Published:** 2025-01-03

**Authors:** Divya S

**Affiliations:** Orthodontics, Manipal College of Dental Sciences, Manipal, Karnataka, India

Dear Sir,

 We have added the references you have mentioned. Also, sample size justification and corrections have been made.

# Reviewer report: Reviewer response for version 1

**DOI:** 10.5256/f1000research.174636.r344500

**Published:** 2024-12-11

**Authors:** Rand Salih Al-Obaidi

**Affiliations:** 1 Mustansiriyah University, Baghdad, Iraq

Dear authors,

 I hope the following comments could help you to improve your manuscript.

 -What are the major differences between Chlorine Dioxide and Chlorhexidine concerning composition, mode of action, concentration..etc?

 -What is the concentration of Chlorine Dioxide used in this study?

 - How did you make the design of your study using CHX mouthwash for 3 months  while it can’t be used for more than one month due to eminent side effects related to prolonged use?

 -I think all figures and tables need more clarification and explanation to make them more evident.

 -The article requires language revision and reformulation.

 -Concerning friction, a greater amount of it for the chlorine dioxide group in comparison with the Chlorhexidine group was seen, but not significantly (p < 0.05).  How can the p < 0.05 and the statistical difference is not significant??  Please check.

 -What is the effect of morphology of arch wires on mouthwash selection? And did you inspect that in your study?

 -(According to the present investigation, ,) I think your findings disagree with your conclusion.

Is the work clearly and accurately presented and does it cite the current literature?

Partly

If applicable, is the statistical analysis and its interpretation appropriate?

I cannot comment. A qualified statistician is required.

Are all the source data underlying the results available to ensure full reproducibility?

No

Is the study design appropriate and is the work technically sound?

No

Are the conclusions drawn adequately supported by the results?

No

Are sufficient details of methods and analysis provided to allow replication by others?

Partly

Reviewer Expertise:

Preventive dentistry and cariology.

I confirm that I have read this submission and believe that I have an appropriate level of expertise to state that I do not consider it to be of an acceptable scientific standard, for reasons outlined above.

# Reviewer report: Reviewer response for version 1

**DOI:** 10.5256/f1000research.174636.r344507

**Published:** 2024-12-02

**Authors:** Ahmed Jasim

**Affiliations:** 1 Al-Mustansiriya University, Al-Rusafa, Iraq

Overall, the article is well-structured and clearly communicates the study's objectives, methods, results, and conclusions.

 Specific Comments:

 Clarity of Objectives: The objectives are clearly stated and focused on the impact of two mouthwashes on the mechanical properties and surface morphology of orthodontic archwires.

 Methodology:

 The experimental design is well-described, including the division into three groups and the number of specimens per group.

 The use of a universal testing machine and atomic force microscopy is appropriate for the study.

 However, more details about brands of wires used and the specific conditions of the immersion (temperature, storage, replenishment of solutions, etc.) would enhance the reproducibility of the study.

 Results:

 The results are presented clearly, including the mean friction and surface roughness values for each group.

 The statistical significance of the differences between the groups is correctly reported.

 Conclusion:

 The conclusion accurately summarizes the main findings of the study.

 It would be helpful to discuss the possible clinical implications of the increased friction, such as potential effects on orthodontic treatment time.

 Additionally, considering the lack of statistical significance in the surface roughness differences, a more nuanced interpretation of the results could be provided.

 Suggestions for Improvement:

 Discussion of Limitations: The research could briefly acknowledge potential limitations of the study, such as the relatively small sample size or the specific brands of mouthwash used.

 Future Directions: Suggesting future research directions, such as investigating the long-term effects of mouthwash exposure or testing a wider range of mouthwash types, could add value to the research.

 By addressing these points, the research can be further strengthened and provide a more comprehensive overview of the study's findings and implications.               

Is the work clearly and accurately presented and does it cite the current literature?

Yes

If applicable, is the statistical analysis and its interpretation appropriate?

I cannot comment. A qualified statistician is required.

Are all the source data underlying the results available to ensure full reproducibility?

Yes

Is the study design appropriate and is the work technically sound?

Yes

Are the conclusions drawn adequately supported by the results?

Yes

Are sufficient details of methods and analysis provided to allow replication by others?

Yes

Reviewer Expertise:

Operative Dentistry, Digital Dentistry, Endodontics, Dental Material

I confirm that I have read this submission and believe that I have an appropriate level of expertise to confirm that it is of an acceptable scientific standard.
